# Non-targeted metabolite profiling of citrus juices as a tool for variety discrimination and metabolite flow analysis

**DOI:** 10.1186/s12870-015-0430-8

**Published:** 2015-02-05

**Authors:** Vicent Arbona, Domingo J Iglesias, Aurelio Gómez-Cadenas

**Affiliations:** Laboratori d’Ecofisiologia i Biotecnologia, Departament de Ciències Agràries i del Medi Natural, Universitat Jaume I, E-12071 Castelló de la Plana, Spain; Institut Valencià d’Investigacions Agràries (IVIA), Moncada, Spain

**Keywords:** Fruit quality, Liquid chromatography, Mass spectrometry, Orange, Phenotyping, Secondary metabolites

## Abstract

**Background:**

Genetic diversity of citrus includes intrageneric hybrids, cultivars arising from cross-pollination and/or somatic mutations with particular biochemical compounds such as sugar, acids and secondary metabolite composition.

**Results:**

Secondary metabolite profiles of juices from 12 commercial varieties grouped into blonde and navel types, mandarins, lemons and grapefruits were analyzed by LC/ESI-QTOF-MS. HCA on metabolite profiling data revealed the existence of natural groups demarcating fruit types and varieties associated to specific composition patterns. The unbiased classification provided by HCA was used for PLS-DA to find the potential variables (mass chromatographic features) responsible for the classification. Abscisic acid and derivatives, several flavonoids and limonoids were identified by analysis of mass spectra. To facilitate interpretation, metabolites were represented as flow charts depicting biosynthetic pathways. Mandarins ‘Fortune’ and ‘Hernandina’ along with oranges showed higher ABA contents and ABA degradation products were present as glycosylated forms in oranges and certain mandarins. All orange and grapefruit varieties showed high limonin contents and its glycosylated form, that was only absent in lemons. The rest of identified limonoids were highly abundant in oranges. Particularly, Sucrenya cultivar showed a specific accumulation of obacunone and limonoate A-ring lactone. Polymethoxylated flavanones (tangeritin and isomers) were absolutely absent from lemons and grapefruits whereas kaempferol deoxyhexose hexose isomer #2, naringin and neohesperidin were only present in these cultivars.

**Conclusions:**

Analysis of relative metabolite build-up in closely-related genotypes allowed the efficient demarcation of cultivars and suggested the existence of genotype-specific regulatory mechanisms underlying the differential metabolite accumulation.

**Electronic supplementary material:**

The online version of this article (doi:10.1186/s12870-015-0430-8) contains supplementary material, which is available to authorized users.

## Background

In the Rutaceae family, citrus constitutes a highly heterogeneous taxonomic group including several species such as sweet oranges (*Citrus sinensis* L. Osbeck), mandarins (*C. clementina* hort. Ex Tan. and *C. reticulata* Blanco), lemons (*Citrus × limon* L. Burm.f.) and grapefruits (*C. paradisi* Macf.). Besides these species, there are other related species with agronomic uses as rootstocks or for ornamental purposes (e.g. *Poncirus trifoliata* L. Raf.). Usually, the different cultivars within a species show low genetic variability but do have particular desirable phenotypic characteristics such as precocity or delayed harvesting, seedless fruits, sugar and acid accumulation, easiness to peel, etc. However, alteration of the harvesting period is one of the most desirable traits, either when precocity or delayed harvesting is achieved. This alteration has additional impacts on fruit quality, as environmental variables change over the year and irradiation, temperature and humidity influence fruit growth, accumulation of sugars and acids and other non-palatable chemical constituents [1-3]. It is difficult to have a control on the buildup of these compounds in fruits over the maturation process. This fraction of citrus juice is constituted, among others, by carotenoids, triterpenoids, flavonoids and other secondary metabolites known to have an impact on health [4,5]. It has been previously shown that different citrus juices have different carotenoid profiles depending on genotype and growth conditions [6] that could have an impact on citrus nutritional properties. To this respect, within a particular growth area, the genotype is expected to be the major contributing factor determining fruit compositional properties, and therefore genetic mutations that give rise to new varieties would also affect fruit chemical composition [7]. Nevertheless, despite the enormous amount of information available it has been so far impossible to establish a reliable model of metabolite flow in citrus fruits. A possible utilization pathway for citric acid was proposed linking it to acetyl-CoA through ATP-citrate lyase after isomerization to isocitrate catalyzed by aconitase [8]. This acetyl-CoA could be in turn channeled to the biosynthesis of secondary metabolites such as limonoids, carotenoids and xanthophylls through the methyl-eriothritiol phosphate pathway. Moreover, biosynthesis of flavonoids and other phenylpropanoids is fueled by intermediates generated during glycolysis and pentose phosphate pathway. To add more complexity to the model, levels of these compounds are determined by the activity of different enzymes that are, in turn, responsible for their biosynthesis, their degradation/biotransformation and/or the conjugation to different chemical moieties. In this sense, as the enzyme activity is generally associated to gene expression, metabolites could be considered the end-products of gene expression [9]. Therefore, to better understand the regulation of secondary metabolism in citrus fruits a comprehensive and unbiased analysis of this class of compounds is required. To this regard, non-targeted LC/MS metabolite profiling has proved to be a valuable tool for phenotyping environmentally- or genetically-induced variations in secondary metabolite composition [10] as well as to evaluate the impact of stress on plant biochemistry [11]. This technique has been previously used to assess adulteration of citrus juice with grape or apple ones [12] and, more recently, to phenotype wild type and mutant orange varieties [7].

The aim of this work was to investigate the differences in secondary metabolite composition within and between five important commercial citrus fruit groups: oranges (blonde and navel), mandarins, grapefruits and lemons (see Table 1). A detailed identification of selected metabolite features was considered in this work to further investigate secondary metabolite flows in every variety, linking diversification to particular metabolite profiles.

**Table 1:** **List of genotypes included in this study**

**#**	**Name**	Species*	Type*	Harvesting period**	°Brix/acidity**
1	Eureka	*Citrus × limon* L. Burm.f.	Lemon	October to February	3-10
2	Fino	*Citrus × limon* L. Burm.f*.*	Lemon	October to April	
3	Marsh	*Citrus paradisi* Macf.	Grapefruit	November to March	4-8
4	Star Ruby	*Citrus paradisi* Macf.	Grapefruit	October to March	
5	Fortune	*Citrus reticulata* Blanco (Clementine mandarin *×* Dancy mandarin)	Mandarin	February to April	8-11
6	Nadorcott	*Citrus reticulata* Blanco (open pollination of Murcott mandarin)	Mandarin	January to March	8-13
7	Pixie	*Citrus reticulata Blanco (open pollination of Kincy mandarin)*	Mandarin	December to February	10-28
8	Hernandina	*Citrus clementina hort. ex Tanaka*	Mandarin	January to February	16-28
9	Sucrenya	*Citrus sinensis L. Osbeck*	Blonde orange	December to March	>40
10	Midknight	*Citrus sinensis L. Osbeck*	Blonde orange	March to June	8-14
11	Washington	*Citrus sinensis L. Osbeck*	Navel orange	December to February	8-13
12	Lane late	*Citrus sinensis L. Osbeck*	Navel orange	January to April	10-16

(*)Information retrieved from University of California, Riverside Citrus variety collection website (http://citrusvariety.ucr.edu). (**) information retrieved from http://www.ivia.es.

## Methods

### Fruit harvesting, sample collection and preparation for analyses

Citrus fruits from different genotypes and varieties (see Table 1) were harvested at commercial maturity from trees at the germplasm bank (Institut Valencià d’Investigacions Agràries, IVIA, Moncada, València). Commercial maturity refers to the timing of harvest to meet specific market and consumer requirements. In citrus, this is assessed by means of the maturity index (°Brix/acidity, see Table 1 for usual maturity index values). Genotypes were characterized according to an enlarged modification of the “Descriptor for Citrus” from the International Plant Genetic Resources Institute (IPGRI) [13]. At least four fruits, one from each direction on the tree, were collected from three replicate trees (n = 3) grafted onto the same rootstock. Juice extraction was performed by manual squeezing and juice of fruits from the same tree was pooled. Juice aliquots were immediately stored at −80°C until analyses with no further processing. Right before chromatographic analyses, frozen fruit juices were thawed at room temperature, centrifuged and the supernatants filtered through PTFE syringe filters (0.2 μm pore size) directly to vials.

### Chromatographic and QTOF-MS conditions

Fruit juices were separated by reversed phase HPLC using acetonitrile (B) and water (A), both supplemented with formic acid to a concentration of 0.1% (v/v), as solvents and a C18 column (5-μm particle size, 100 9 2.1 mm, XTerra™, Waters). The separation module, a Waters Alliance 2965 was operated in gradient mode at a flow rate of 300 μl min^−1^ for 30 min as follows: 0–2 min 95:5 (A:B) followed by an increase in B from 5 to 95 in the following 26 min (2.01-28.00 min), thereafter returning to initial conditions (29.01-30.00 min) that were maintained for 5 min for column reconditioning. Column eluates were introduced into a QTOF-MS (Micromass Ltd., Manchester, UK) through an ESI source operated in positive and negative mode. Nitrogen was used as the nebulization as well as the desolvation gas and working flows were set at 100 and 800 L h^−1^, respectively. Source block temperature was kept at 120°C and desolvation gas at 350°C. Capillary, cone, and extractor voltages were set at 4 kV, 25 eV, and 3 eV, respectively. Before analyses, the QTOF-MS was calibrated by infusing a mixture of NaOH and HCOOH at a flow rate of 25 μl min^−1^. After calibration, the average error was less than 5 ppm. During acquisition, a one-ppm solution of Leu-enkephalin ([M+H]^+^ = 556.2771) was continuously post column infused as a lockmass reference. Data were acquired under continuous mode in the 50–1000 amu range, scan duration was set at 1.0 s, and interscan delay was set at 0.1 s.

### Data processing

Data processing was achieved using Masslynx v.4.1 and raw data files were analyzed using xcms following conversion to netCDF with the databridge software provided by Masslynx. Chromatographic peak detection was performed using the matchedFilter algorithm, applying the following parameter settings: snr = 3, fwhm = 15 s, step = 0.01 D, mzdiff = 0.1 Da, and profmethod = bin. Retention time correction was achieved in three iterations applying the parameter settings minfrac = 1, bw = 30 s, mzwid = 0.05 Da, span = 1, and missing = extra = 1 for the first iteration; minfrac = 1, bw = 10 s, mzwid = 0.05 Da, span = 0.6, and missing = extra = 0 for the second iteration; and minfrac = 1, bw = 5 s, mzwid = 0.05 Da, span = 0.5, and missing = extra = 0 for the third iteration. After final peak grouping (minfrac = 1, bw = 5 s) and filling in of missing features using the fillPeaks command of the xcms package, a data matrix consisting of mass features (including accurate mass values and retention time) and peak area values per sample was obtained.

### Statistical analyses

**Figure 1:**
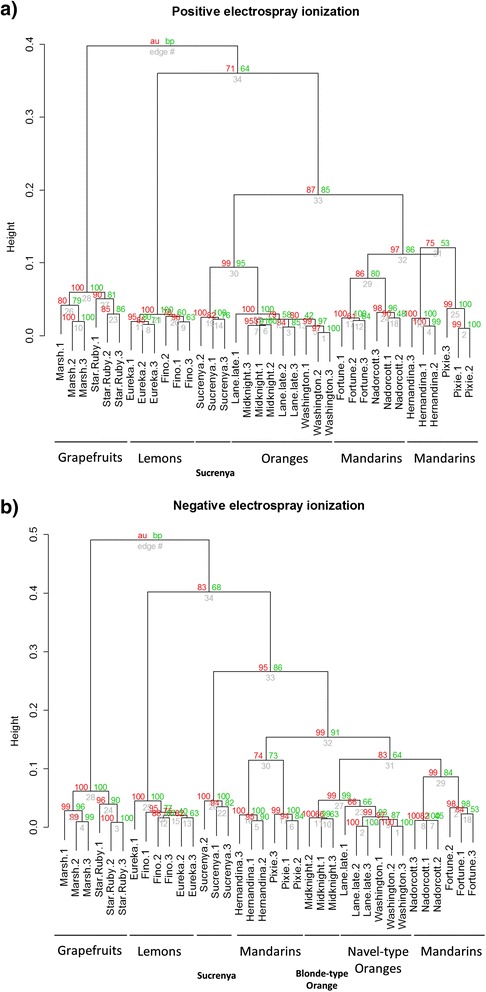
**Hierarchical clustering dendrograms obtained from (a) positive and (b) negative electrospray metabolite profiles of citrus juices.** On every node, approximate unbiased (red, au) and bootstrap values (green, bp) are presented.

Hierarchical Cluster Analysis (HCA) was performed with pvclust package running under R 3.2 and PLS-DA was performed using SIMCA-P+ 11.0 (Umetrics, Umea, Sweden). HCA, followed by bootstrap resampling (n = 1000) to validate grouping, was performed on raw data without any variable selection to observe natural grouping of samples. The classification provided by unsupervised HCA confirmed homogeneity of sample groups (Figure 1) and allowed using genotype denomination as parameter to feed PLS-DA. This strategy was further used to select potential variables contributing to the provided classification. Prior to analyses, data were normalized to total ion intensity. The potential variables contributing to the classification were selected based on variable importance in the projection (VIP > 2.0) values. Relevant variables were then confirmed after integration of chromatographic peaks and analysis of variance (ANOVA) of peak areas throughout the 12 sample groups. The metabolites were tentatively identified by elucidation of structures with MS fragments, comparison of accurate *m/z* value and MS fragmentation pattern with literature and co-injection with pure standards when available. All standards were purchased from Sigma-Aldrich (Madrid, Spain) except for ABA and derivatives that were obtained from the Plant Biotechnology Institute of the National Research Council (Canada).

## Results and discussion

### Non-targeted analysis of secondary metabolite features in citrus fruit juices

The analyses, carried out by means of reversed-phase liquid chromatography coupled to a QTOF-MS operated in positive and negative ionization modes, rendered a number of chromatograms that were extracted with XCMS [14]. The resulting datasets were subjected to HCA using the R package pvclust and presented as dendrograms in Figure 1. The results showed grouping of sample replicates in tight clusters according to the juice source fruit (see Table 1). In addition, relationship between clusters was in agreement with the expected phylogenetic relationships among varieties showing a perfect separation of the represented groups: grapefruits, lemons, oranges (blonde and navel types) and mandarins (see Additional file 1: Figure S1). All varieties could be resolved using different component combinations after PLS-DA. In addition, loadings plots indicated that some variables were important in defining the different sample groups (Additional file 2: Figure S2a through f). Component 2 resolved well ‘Washington’ navel from the rest whereas ‘Sucrenya’ resolved along component 3. A combination of components 5 and 6 allowed the resolution of grapefruits and the two varieties included in this group. Component 5 alone allowed the discrimination of ‘Hernandina’ from the rest of varieties. A better resolution for grapefruits was obtained along component 8. Meanwhile, component 7 resolved well ‘Nadorcott’ and ‘Midknight’ varieties. Lemons resolved along component 10 whereas component 9 discriminated ‘Pixie’ from the rest. A combination of components 9 and 10, allowed demarcation of ‘Fortune’ and ‘Lane late’ although these two varieties were better resolved along component 11 in combination with component 1 (Additional file 2: Figure S2f). The two grapefruit varieties were the utmost distant species included in the study followed by lemons, both constituting highly tight clusters in the HCA (Figure 1). This is probably due to their clear phylogenetic origin, grapefruits are crosses between sweet orange *Citrus sinensis* and *Citrus maxima* (pummelo), whereas lemons arise from the cross of sour orange *Citrus aurantium* and *Citrus medica* (citron, see Additional file 1: Figure S1 for more details). Two major clusters originated from grouping oranges (‘Sucrenya’, ‘Lane late’, ‘Midknight’ and ‘Washington’) and mandarins (‘Hernandina’, ‘Pixie’, ‘Fortune’ and ‘Nadorcott’) that are also phylogenetically related. To this respect, although ‘Lane late’, ‘Midknight’ and ‘Washington’ always occurred together, ‘Sucrenya’ appeared as a separate cluster probably due to its acidless juice characteristics. Moreover, in both ionization modes the methodology efficiently demarcated mandarins in two groups: ‘Fortune’/‘Nadorcott’, arising from clementine × mandarin cross-pollination and an open pollination of ‘Murcott’ mandarin (see Table 1) respectively,and ‘Hernandina’/‘Pixie’, resulting from a bud mutation from ‘Fina’ clementine and an open pollination of ‘Kincy’ mandarin, correspondingly. Mandarins are self-incompatible citrus species that usually produce seedless fruits unless flowers are cross-pollinated with compatible species. These cross-pollination has been extensively used to generate new commercial cultivars with particular fruit traits that differ from those of each parental. Examples of this are ‘Fortune’ and Nadorcott’, often classified as mandarin hybrids, which share several fruit morphology, color and aroma characteristics. On the other hand, ‘Hernandina’ and ‘Pixie’, although are classified as two different species, they show more similar phenotypic traits, including morphology, flavor and period of maturation [15]. It is likely that despite of differences in their genetic origin the respective overcrosses yielded varieties with similar metabolite phenotypes quite different from the rest of varieties included in this study. Profiling of citrus juices in negative electrospray also gave the required resolution to discriminate genotypes included in the navel and blonde groups: ‘Lane late’/‘Washington’ and ‘Sucrenya’/‘Midknight’, respectively. In this sense, it is worthwhile to note that ‘Sucrenya’ always occurred as a separate group from oranges. This is likely a result of its particular juice traits. This variety usually shows very low titratable acid contents, compared to the rest of blonde or navel-type varieties [8]. Nevertheless, although all related varieties (within the same group) clustered together, it was still possible to clearly differentiate each of them (Figure 1).

### Variable selection and annotation of compounds

**Figure 2:**
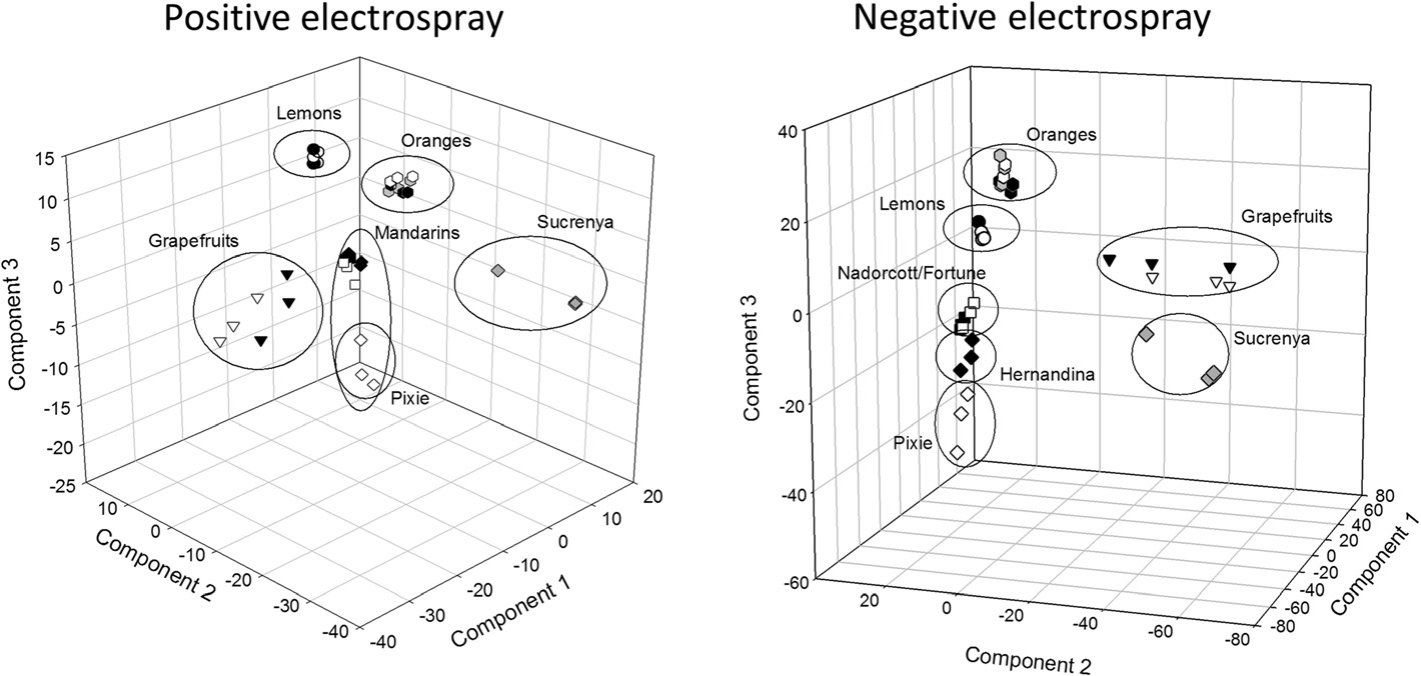
**Scores 3D scatter plots after PLS-DA analysis.**

In order to identify those variables contributing to the observed classification (Figure 2), a PLS-DA was carried out using the entire XCMS output using sample classification provided by HCA. PLS-DA calculates a regression model between the multivariate dataset (each variable consisting of a *m/z* and retention time value) and a response variable that only contains class information (e.g. the variety classification provided by HCA). This analysis yielded a number of variables (chromatographic peaks, each represented by *m/z* and retention time values) ranked from very important (VIP > 2 to 1.5) to irrelevant (VIP values lower than 1). Scores 3D scatter plots from PLS-DA results indicated an optimal performance of the model to differentiate big groups of fruits: lemons, grapefruits, oranges and mandarins (Figure 2) and, in addition, some varieties were clearly differentiated within their respective groups such as both grapefruit cultivars, ‘Pixie’ mandarin and ‘Sucrenya’ blonde orange. Nevertheless, by representing other combinations of components the model is also able to clearly differentiate closely-related varieties within a group (data not shown). In general, varieties were grouped according to genotype and not harvesting period (Table 1). It seems clear that environmental growth conditions have an influence on fruit secondary metabolite composition as shown in [6]. In that case, carotenoid composition of orange and mandarin varieties grown in Mediterranean, subtropical and tropical conditions was evaluated showing clear differences. However, when the same parameter was evaluated in varieties grown in the same climatic conditions, little changes could be observed throughout the year. Therefore, the differences in secondary metabolite composition observed in the present work are likely to arise as particular genotype traits rather than being induced by environmental changes. Biochemical evolution of fruits throughout the ripening process is also an important aspect. In this work, all fruits were harvested at optimum commercial maturity. It is likely that fruit metabolite composition changes during fruit growth and maturation and also during the postharvest period. However, it is expected that they keep their characteristic traits. Recently, it was shown that even after industrial orange juice processing it was possible to identify adulteration with other juice sources, such as apple or grapefruit [12]. This demonstrates that industrial juice processing is not sufficient to remove or mask the discriminant metabolite of orange juice. Moreover, metabolomic analysis of pulp extracts of an orange bud mutant variety and its parental at different harvesting dates revealed higher differences between varieties than among sampling dates. Therefore, it could be hypothesized that differences among varieties could be minimized throughout the ripening process; however, the discriminant metabolite traits allowing demarcation of genotypes would still remain present.

**Figure 3:**
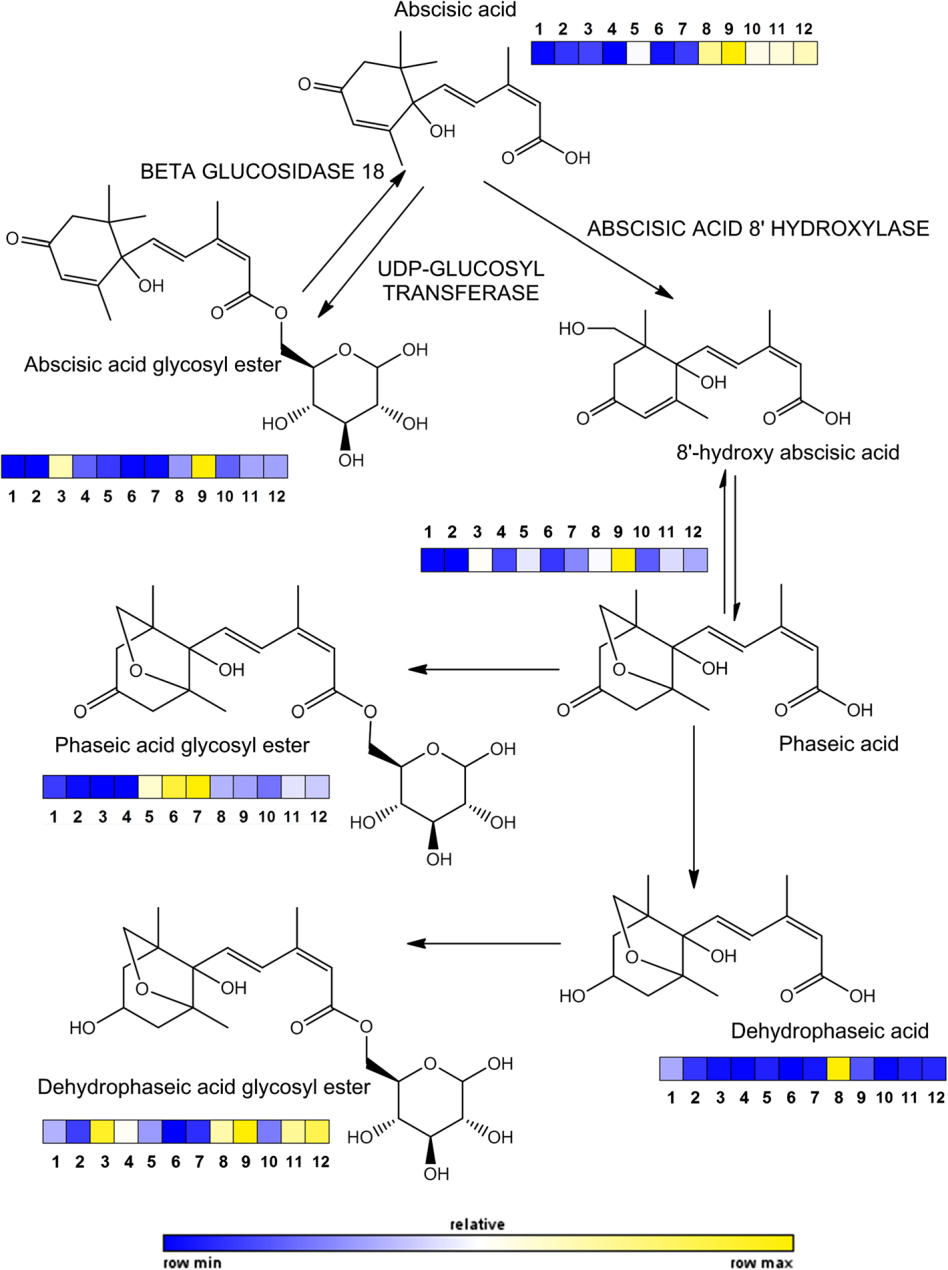
**Scheme of ABA metabolism, including chemical structure of free and conjugated forms and products of degradation.** On every compound a color scale indicates relative amounts in juices of each variety studied. Sample ID followed the same order as in Table 1.

**Figure 4:**
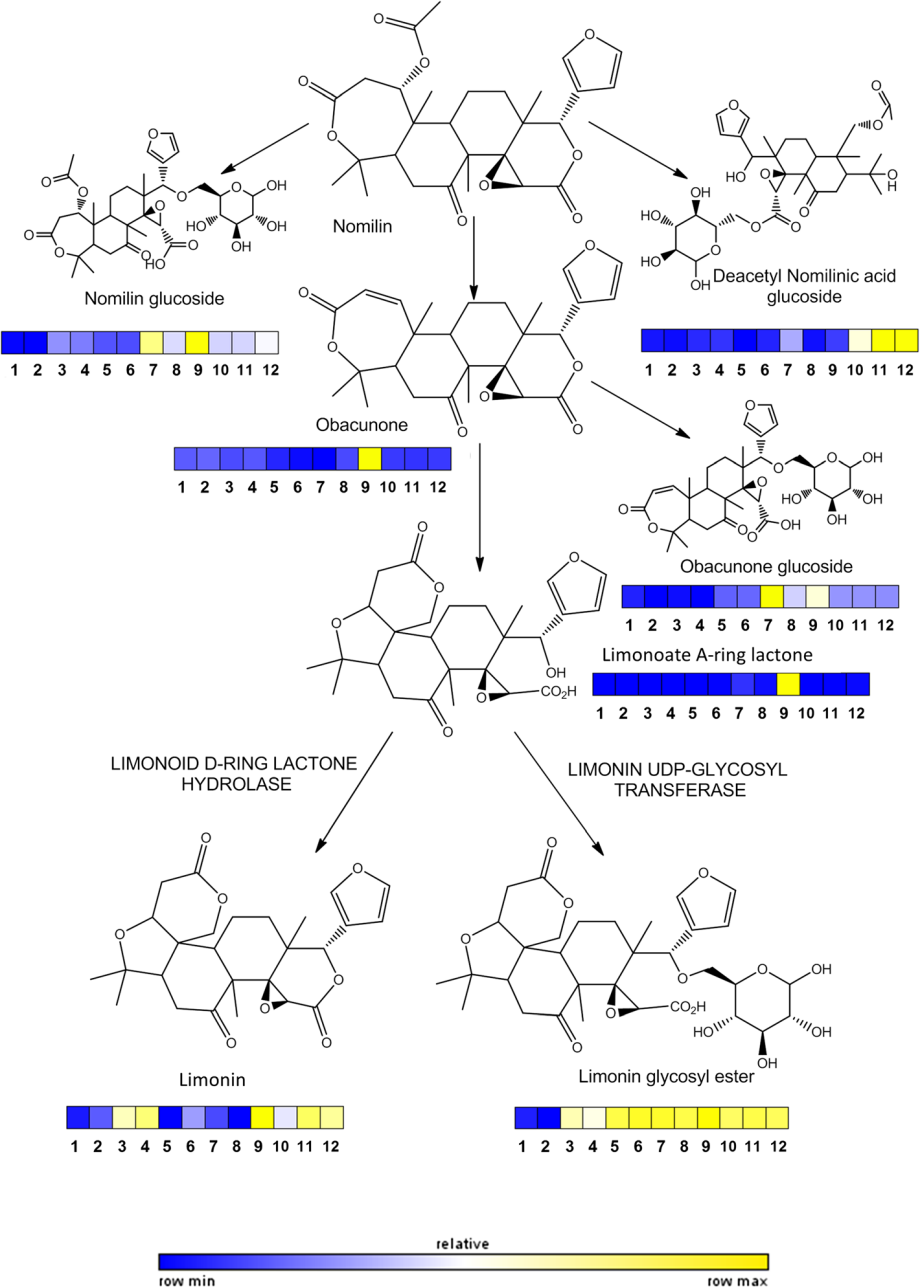
**Scheme of limonoid metabolic pathway arising from nomilin.** On every compound a color scale indicates relative amounts in juices of each variety studied. Sample ID followed the same order as in Table 1.

**Figure 5:**
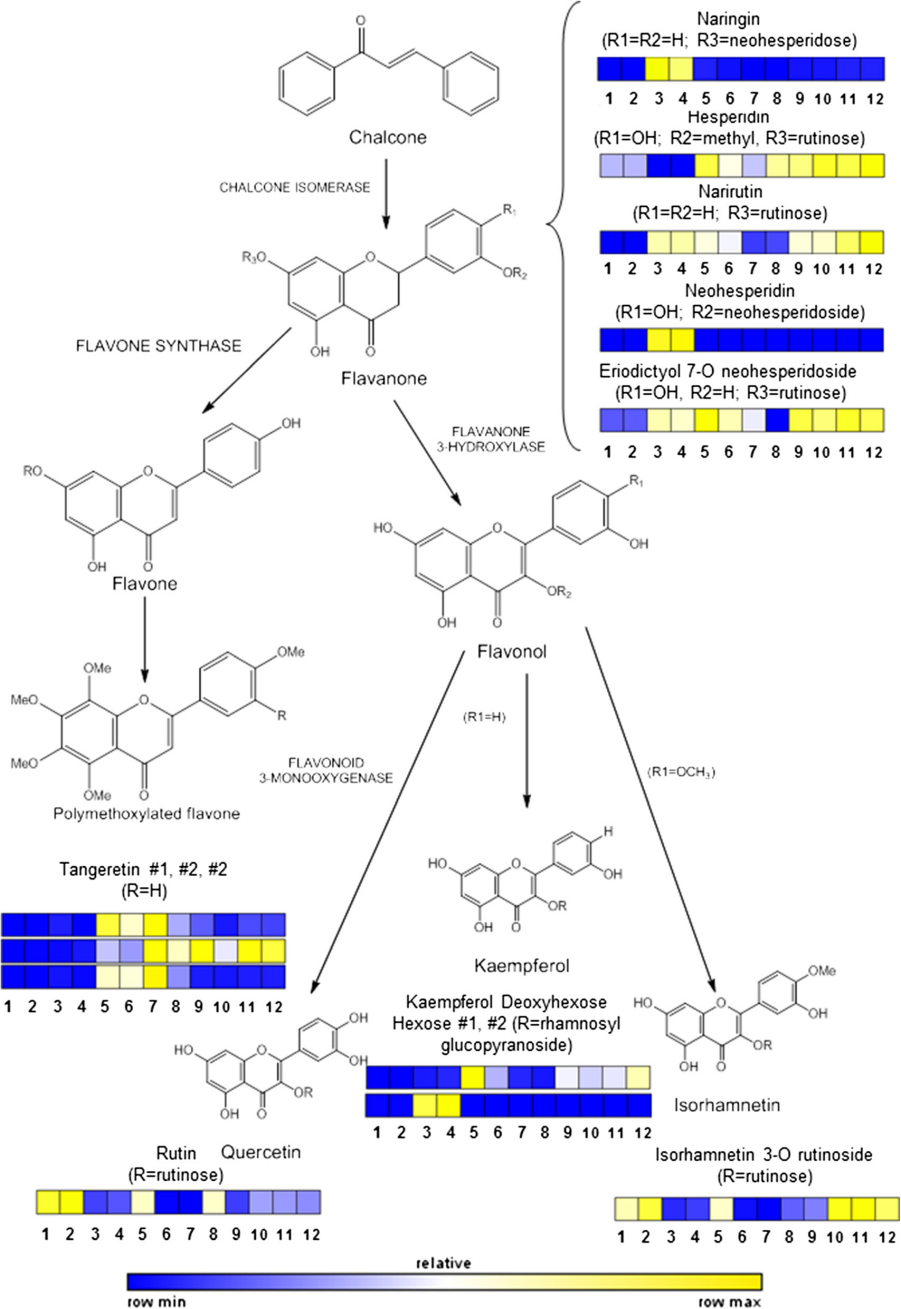
**Scheme of flavonoid metabolic pathway arising from chalcone (not analyzed).** On every compound a color scale indicates relative amounts in juices of each variety studied. Sample ID followed the same order as in Table 1.

Chromatographic mass features showing a VIP value higher than 1.5 were located and further inspected using Masslynx 4.1. software to attain structure elucidation and annotation of compounds. A number of potential metabolites were identified and annotated based on structural elucidation, literature search and comparison with commercial standards, when available (Table 2). According to their putative annotation, all compounds were grouped into metabolite classes and their relative accumulation represented as metabolite flow charts (Figures 3, 4 and 5). ABA and its derivatives were identified based on mass spectra and/or comparison with commercial standards. It has been previously shown that variations in the expression of *NCED2* and *3* are correlated with endogenous ABA levels. To this respect, juice sacs of satsuma mandarin had higher ABA levels than those of lemons or sweet oranges along with higher NCED expression [16]. This could be somehow associated to differences found in carotenoid content among citrus varieties [6]. Besides changes in expression and activity of NCEDs, carotenoid precursor availability could influence ABA content. Citrus fruits are also important sources of flavonoids, including several kaempferol, hesperetin, naringenin and isorhamnetin derivatives that were putatively identified based on the literature and the comparison with commercial standards. In addition, three metabolites showing a *m/z* compatible with their annotation as tangeretin ([M+H]^+^ 373.1397, ΔDa −0.011) were detected under positive electrospray ionization (Table 2). This would indicate the presence of different tangeretin isomers with identical composition but methoxylated in different positions. Moreover, some limonoids were annotated in citrus samples. These compounds are triterpenoids derived from squalene by formation of a polycyclic molecule containing a furanolactone core structure [17] and some of them are known to provide bitter taste to citrus juices namely limonin, nomilin, obacunone and nomilinic acid. Limonoids can also be released from their respective glycosylated forms upon cleavage after freeze damage or other environmental stress conditions [18]. These compounds have been associated to fruit quality and reported to have important health benefits [17,19,20]. Besides, some bitter limonoids can be present as tasteless A-ring lactones that were also tentatively annotated in this work. In addition, some compounds involved in other mixed pathways, such as the aminoacids Phe and Trp, involved in aromatic and indolic compound biosynthesis [21,22] and a ferulic acid hexoside, derived from the phenylpropanoid pathway, were also annotated.

**Table 2:** **Identification of compounds**

**Compound**	**ESI +**	**annotation positive**	**ESI -**	**annotation negative**	**Rt (min)**	**Rt (s)**	**annotation level**	**ChEBI code**
*Abscisic acid and derivatives*								
Dihydrophaseic acid glycosil ester (DPAGE)	**247.1445**	[M+H-H_2_O]^+^	**443.1941**	[M-H]^−^	4.15	249.3	2, 3 [23]	
	265.1483	[M+H-Glucose]^+^	479.1836	[M+Cl]^−^				
	467.2023	[M+Na]^+^	489.1993	[M+HCOOH]^−^				
	483.1752	[M+K]^+^						
Phaseic acid glycosyl ester (PAGE)	**247.1447**	[M+H-Glucose-H_2_O]^+^	**442.1841**	[M-H]^−^	5.83	350.0	1, 3	
	467.2059	[M+Na]^+^						
	483.1753	[M+K]^+^						
Dihydrophaseic acid (DPA)	**265.1454**	[M+H-H_2_O]^+^	**281.1455**	[M-H]^−^	8.46	507.6	2, 3 [23]	CHEBI:23757
	247.1357	[M+H-2 × H_2_O]^+^						
	305.1456	[M+Na]^+^						
Abscisic acid glycosyl ester (ABAGE)	229.1329	[M+H-H_2_O]^+^	**425.1833**	[M-H]^−^	10.26	615.6	1	CHEBI:62436
	247.1379	[M+H-Glucose]^+^	471.1915	[M-H+HCOOH]^−^				
	**265.1528**	[M+H-Hexose]^+^	263.1404	[M-Hexose]^−^				
	449.1775	[M+Na]^+^						
	465.1740	[M+K]^+^						
Phaseic acid (PA)	**247.1357**	[M+H-2 × H_2_O]^+^	**279.1400**	[M-H]^−^	11.56	693.6	1	CHEBI:28205
	265.1490	[M+H-H_2_O]^+^						
	229.1490	[M+H-3 × H_2_O]^+^						
Abscisic acid (ABA)	**247.1385**	[M+H-H_2_O]^+^	**263.1374**	[M-H]^−^	12.52	751.2	1	CHEBI:2365
	303.1071	[M+K]^+^						
	265.1495	[M+H]^+^						
	328.1577	[M+Na+CH_3_CN]^+^						
*Limonoids and glycosides*								
Limonin glycoside	**471.2049**	[M+H-Glucose]^+^	**649.2438**	[M-H]^−^	10.20	612.0	2, 3 [24]	CHEBI:16063
	673.2702	[M+Na]^+^						
	689.2392	[M+K]^+^						
	489.2241	[M+H-Hexose]^+^						
Deacetyl Nomilinic acid glycoside	nd		**669.2733**	[M-H]^−^	10.55	633.0	2, 3 [24]	
Limonin A-ring lactone*	**471.2007**	[M+H-H_2_O]^+^	**487.1945**	[M-H]^−^	10.93	655.8	2, 3 [24]	CHEBI:16226
Deacetyl Nomilin glycoside	nd		**651.2624**	[M-H]^−^	11.08		2, 3 [24]	
Nomilinic acid glucoside	nd		**711.2627**	[M-H]^−^	11.82	709.2	2, 3 [24]	
Nomilin glycoside	**515.2332**	[M+H-Glucose]^+^	**693.2737**	[M-H-H_2_O]^−^	11.87	712.2	2, 3 [24]	
	533.2402	[M+H-Hexose]^+^	711.2837	[M-H]^−^				
	455.2494	[M+H-CH4O]^+^						
	695.2495	[M+H]^+^						
	487.2391	[M+H-CO]^+^						
	419.2000	[M+H-2xH_2_O]^+^						
Obacunone glycoside	**531.3160**	[M+H-C_4_H_8_O_3_]^+^	**633.2513**	[M-H]^−^	12.25	735.0	2, 3 [24]	
	455.2069	[M+H-Glucose]^+^						
Nomilin A-ring lactone*	**533.2700**	[M+H-H_2_O]^+^	**531.2330**	[M-H]^−^	14.75	885.0	2, 3 [24]	
Limonin	**471.2031**	[M+H]^+^	515.1922	[M+HCOOH]^−^	16.60	996.0	1	
	427.2233	[M-CO_2_]^+^	**469.1904**	[M-H]^−^				
	512.2452	[M+CH_3_CN]^+^	505.1670	[M+Cl]^−^				
Nomilin	**515.2448**	[M+H]^+^	**513.2211**	[M-H]^−^	17.50	1050.0	2, 3 [24]	
	455.2251	[M+H-C_2_H_4_O_2_]^+^						
Obacunone	nd		**453.2200**	[M-H]^−^	21.38	1282.8	2, 3 [24]	
*Flavonoids*								
Eriodictyol 7-O rutinoside	**595.1725**	[M+H]^+^	**593.1387**	[M-H]^−^	8.92	535.2	2, 3 [25]	
	451.0975	[M+H-Deoxyhexose]^+^	449.1096	[M-H-Deoxyhexose]^−^				
	289.0905	[M+H-Hexose-Deoxyhexose]^+^						
Rutin	**611.1700**	[M+H]^+^	**609.1772**	[M-H]^−^	10.25	615.0	1	CHEBI:28527
	449.1563	[M+H-Hexose]^+^						
	303.0947	[M+H-Hexose-Deoxyhexose]^+^						
Narirutin	**581.1946**	[M+H]^+^	**579.1660**	[M-H]^−^	10.82	649.2	1	CHEBI:28705
	419.1390	[M+H-Hexose]^+^	615.1440	[M+Cl]^−^				
	273.0783	[M+H-Hexose-Deoxyhexose]^+^	271.0668	[M-H-Hexose-Deoxyhexose]^−^				
	435.1369	[M+H-Deoxyhexose]^+^						
	401.1318	[M+H-Glucose]^+^						
	603.1908	[M+Na]^+^						
Isorhamnetin-3-O-rutinoside	**625.1985**	[M+H]^+^	**623.1828**	[M-H]^−^	10.86	651.6	2, 3 [25]	
	317.0667	[M+H-Rutinose]^+^						
	479.1347	[M+H-Deoxyhexose]^+^						
Naringin	**581.1829**	[M+H]^+^	**579.1614**	[M-H]^−^	11.14	668.4	1	CHEBI:28819
	435.1303	[M-Hexose]^+^						
	419.1327	[M-Hexose-H_2_O]^+^						
	273.0775	[M+H]^+^						
Hesperidin	**611.1993**	[M+H]^+^	**609.1722**	[M-H]^−^	11.27	676.2	1	CHEBI:28775
	449.1449	[M-Hexose]^+^	301.0767	[M-Hexose-Deoxyhexose]^−^				
	303.0947	[M-Hexose-Deoxyhexose]^+^	279.1298					
	495.1524	[M+H-C_5_H_8_O_3_]^+^						
Neohesperidin	**611.2115**	[M+H]^+^	**609.1772**	[M-H]^−^	11.53	691.8	2, 3 [25]	CHEBI:59016
	449.1539	[M-Hexose]^+^						
	303.0948	[M-Hexose-Deoxyhexose]^+^						
Kaempferol Deoxyhexoside Hexoside #1	**595.1475**	[M+H]^+^	**593.1871**	[M-H]^−^	12.93	775.8	2, 3 [25]	
	433.1568	[M+H-Hexose]^+^	639.1884	[M+HCOOH]^−^				
	287.1010	[M+H-Hexose-Deoxyhexose]^+^						
Kaempferol Deoxyhexoside Hexoside #2	**287.0959**	[M+H-Deoxyhexose-Hexose]^+^	**593.1887**	[M-H]^−^	13.20	792.0	2, 3 [25]	
	595.2134	[M+H]^+^	639.1846	[M+HCOOH]^−^				
	433.1555	[M-Hexose]^+^						
	449.1511	[M-Deoxyhexose]^+^						
Tangeretin #1	**373.1397**	[M+H]^+^	nd		15.04	902.4	3	CHEBI:9400
	436.1960	[M+NaCH_2_CN]^+^						
Tangeretin #2	**373.1397**	[M+H]^+^	nd		15.93	955.8	3	CHEBI:9400
	436.1960	[M+NaCH_2_CN]^+^						
	411.1044	[M+K]^+^						
	395.1297	[M+Na]^+^						
Tangeritin #3	**373.1397**	[M+H]^+^	nd		18.14	1088.4	3	CHEBI:9400
	436.1960	[M+NaCH_2_CN]^+^						
	411.1044	[M+K]^+^						
	395.1297	[M+Na]^+^						
*Miscellaneous compounds*								
Phenylalanine	**166.0693**	[M+H]^+^	nd		2.10	126.0	1	CHEBI:17295
	120.0588	[M+H-NH_3_]^+^						
Tryptophan	205.1006	[M+H]^+^	nd		4.56	273.6	1	CHEBI:16828
	**188.0719**	[M+H-NH_3_]^+^						
	144.0951	[M+H-NH_3_-CO_2_]^+^						
Ferulic acid hexoside	**379.1035**	[M+Na]^+^	**355.0981**	[M-H]^−^	8.10	486.0	1, 3	
	177.0488	[M+H-Hexose-H_2_O]^+^						
	195.0595	[M+H-Hexose]^+^						
	395.0840	[M+K]^+^						

Annotation level: 1) co-injected with pure standards, 2) annotated matching published data and mass spectral results and 3) annotation made based on mass spectral data, *) tentatively annotated and nd) not determined. *m/z* values in bold are quantifier ions.

For an easier interpretation of data, flow charts depicting biosynthetic pathways (constructed according to the current information available on Kegg, http://www.genome.jp/kegg/) are presented in this work. This allows classifying most metabolites as part of specific biosynthetic pathways, the relative concentration of each metabolite throughout all analyzed genotypes is represented as a color scale (Figures 2, 3 and 4) following the same sample order as in Table 1. The validity of each metabolite marker was assessed by ANOVA comparing peak areas throughout sample groups (Additional file 3: Table S1). This was achieved using the quantifier ion (an ion with the highest intensity within the spectrum of a given metabolite, marked in bold in Table 2) to extract metabolite peaks with Masslynx 4.1. software.

### ABA and derivatives

The pathway, starting from ABA, has two major branches: the catabolic and the conjugating branch. The first one starts with the conversion of ABA into 8′-hydroxy ABA (catalyzed by ABA 8′-hydroxylase), which spontaneously isomerizes to PA. This metabolite is further catabolized to DPA by a soluble reductase [26]. The conjugating branch involves the temporary storage of ABA into a glycosylated form catalyzed by an UDP-ABA glycosyl transferase (Figure 3). The most widespread form is ABAGE which is the result of esterification at the C1 position of the carboxyl group [26,27]. In turn, active ABA can be released from ABAGE by a glycosidase (BGLU18, [28]).

ABA levels in fruits of the ‘Sucrenya’ orange were the highest. Whereas, high contents of this hormone were also found in ‘Hernandina’, ‘Midknight’, ‘Washington’, and ‘Lane late’; and ‘Fortune’, ABA levels were much lower in lemons, grapefruits and Pixie and Nadorcott mandarin cultivars. In general, varieties showing low ABA content had also low concentrations of ABA catabolites, including ABAGE (Figure 3). Conversely, ‘Sucrenya’ that showed the highest ABA levels had also the highest PA and ABAGE levels among all varieties. These results suggested a different ABA metabolic fingerprinting for each variety. ABA levels seem to be regulated by degradation to DPA followed by conjugation in ‘Hernandina’. On the other hand, ABA metabolism in ‘Nadorcott’ and ‘Pixie’ as well as in ‘Lane late’ and ‘Washington’ oranges appeared to be channeled to the production of glycosylated forms of PA and DPA, respectively, showing scarce accumulation of their free forms. Surprisingly, the other blonde-type variety, ‘Midknight’, did not accumulate any catabolite or ABA derivative, suggesting that the control of ABA levels took place by regulating its biosynthesis (NCED activity). On the contrary, in Fortune ABA levels appeared to be regulated in by diverting metabolic flow to PA and PAGE synthesis. The rest of cultivars accumulating low ABA contents such as lemons a general downregulation of the pathway was found whereas in grapefruits metabolite flow was directed to DPAGE synthesis (with a particular behavior of Marsh genotype that accumulated significant amounts of PA and ABAGE). Noteworthy, only ‘Sucrenya’ orange and ‘Marsh’ grapefruit showed significantly higher ABAGE levels than the rest of varieties. Overall, this indicates that citrus fruits and especially juice sacs preferentially induce the degradation pathway to reduce ABA levels (being conjugation of ABA a less relevant mechanism). In previous reports, higher ABA levels were found in juice sacs of satsuma mandarin (*Citrus unshiu*) compared to ‘Lisbon’ lemon or ‘Valencia’ orange [16]. This could be explained in part by a higher ability of satsuma mandarin for carotenoid and xanthophyll biosynthesis in juice sacs together with a higher metabolite flow towards xanthoxin and ABA [29]. On the contrary, although carotenoid availability in mandarins is higher than in oranges [15], it is likely that availability of xanthophyll substrates needed for NCED activity is much lower probably channeling these precursors to other metabolic pathways, thus contributing to lower ABA levels in this group (Figure 3). Nevertheless, in ‘Nadorcott’ and ‘Pixie’ cultivars, increased degradation to PA along with its conjugation to hexoses rendering PAGE could also contribute to decreased ABA levels. In contrast, the lemon and grapefruit varieties showed lower levels of ABA and catabolites and observation coincident with the reported low carotenoid levels in juice sacs [30].

### Limonoids

Limonoids are highly oxygenated triterpenes present in Rutaceae and Meliaceae. These compounds are derived from squalene, although the first true limonoid precursor is nomilin that can be directly glucosylated by a limonoid UDP-glucosyl transferase or also deacetylated (Figure 4) rendering obacunone. Cleavage of C-O bond at the D-ring and reorganization of the D-ring renders the tasteless limonoate A-ring lactone that can be alternatively glycosylated (as occurs during normal maturation) or converted into bitter limonin [17]. All identified limonoids were present in all varieties at different levels but especially in lemons showed very low values (Figure 3). Particularly, limonin glycosyl ester was present at similar levels in all orange and mandarin varieties but showed slightly lower levels in the two grapefruit varieties. These two genotypes, together with orange cultivars, also contained high concentrations of the bitter limonin whereas mandarins showed very low values. In addition, ‘Sucrenya’ was the only variety that had significant amounts of limonoate A-ring lactone and obacunone, suggesting a highly active biosynthesis in this variety. Nomilin could not be detected in this study but its glycosylated and deacetylated derivatives (Figure 4 and Table 2). Nomilin glucoside levels were much higher in ‘Sucrenya’ and ‘Pixie’ cultivars than in the rest of citrus varieties that showed very low levels. Levels of Deacetylated nomilin glucoside in the navel-type oranges (‘Washington’ and ‘Lane late’) were the highest whereas they were slightly lower in ‘Midknight’ and in trace amounts in ‘Pixie’. Obacunone glucoside levels were high in this variety and lower levels, by decreasing order, were detected in ‘Sucrenya’, ‘Hernandina’ and the rest of oranges. This indicated that all limonoid pool was diverted into production of glycosides, as expected in normal maturation, but some varieties also presented significant amounts of bitter limonin [31], including lemons, ‘Sucrenya’, ‘Midknight’ and navel-type oranges. Nomilin glucoside, obacunone, obacunone glucoside, limonoate A-ring lactone and its glucoside and limonin are over-accumulated in ‘Sucrenya’ compared to the other blonde-type variety ‘Midknight’. This suggests a particularly active limonoid biosythetic pathway in ‘Sucrenya’ whereas in ‘Midknight’ all intermediates are readily channeled to the production of deacetyl nomilic acid glucoside, limonin and limonin glucoside. Indeed, limonin glucoside was highly abundant in almost all studied citrus juices (0.035% of juice weight in mexican lime, as described in [19]) that could also be cleaved to render free limonin upon induction of a glucosidase [32]. The concentration of limonoid metabolites highly increased in citrus affected by bacterial Greening Disease or Huanglongbing (HLB) [33] suggesting a role in defense against bacterial infection. Moreover, limonoids have exhibited significant antioxidant and antitumorigenic activity [19,20]. However, their specific physiological role in citrus is still unknown. The results presented here also point out differences in palatable fruit quality among varieties at optimum commercial maturation stage, likely associated to genetic differences.

### Flavonoids

This class of compounds has been involved in the antioxidant and beneficial health properties of citrus. Indeed, the high radical scavenging activity of citrus juices has been almost exclusively associated to flavonoids and other phenolic constituents [19]. In citrus, the most abundant flavonoids are flavanones, flavones and flavonols being the methoxylation and glycosylation the main reactions rendering derivatives [34]. In this study, from the same flavanone core several derivatives were identified by substitution with methyl groups or hexose moieties: naringin, hesperidin, narirutin, neohesperidin, and eriodictyol (Figure 5 and Table 2). From this group, the most widespread compounds were hesperidin, narirutin and eriodictyol 7-O-neohesperidoside, whereas naringin and neohesperidin were exclusively present in grapefruits. Flavonoid synthesis starts from the flavanone naringenin by successive transfer of glycosyl groups (a first step by which glucose is transferred to oxygen in position 7 generating a 7-O-glucoside). In turn, a 1,6 rhamnosyl transferase renders the hesperidosides (or rutinosides) hesperidin and narirutin. Conversely, action of 1,2 rhamnosyl transferase on flavanone 7-O-hexosides generates neohesperidosides: neohesperidin and naringin. A very low expression of 1,6 rhamnosyl transferases in citron, pummelo and grapefruit and absence of 1,2 rhamnosyl transferases in mandarins and oranges have been recently reported [35]. These results would explain the exclusive occurrence of neohesperidosides in grapefruit cultivars in the present work (Figure 4). Apparently, this low expression is enough to grant occurrence of rutinosides such as narirutin and eriodictyol 7-O rutinoside in grapefruits. Another group of flavonoids, flavonols, synthesized from the same flavanones by hydroxylation include isorhamnetin, kaempferol and quercetin. Rutin showed the highest accumulation in lemons, although it was present in most citrus cultivars included in this study, showing significantly lower levels in ‘Fortune’ and ‘Hernandina’. This could point out a higher flavonoid 3-monooxygenase activity in these genotypes. Isorhamnetin 3-O rutinoside derived from addition of an hexose moiety on oxygen in position 3 catalyzed by 3-glycosyl transferase followed by 1,6 rhamnosyl transferase [35]. It is now clear that 1,6 rhamnosyl transferase is present and active to different levels in most cultivated citrus species. Therefore, the selectivity relies on the previous action of 7- or 3-glycosyl transferases. To this respect, the results obtained suggest that 3-glycosyl transferases are likely to be rather active in grapefruit cultivars, therefore rendering flavonol 7-O rutinosides. Isorhamnetin 3-O-rutinoside, product of methoxylation and subsequent glycosylation of a flavonol moiety was found to be highly abundant in both navel oranges and ‘Midknight’ and in the two lemon cultivars which could point out at flavonol A-ring methoxylation being highly active in these genotypes. On the other hand, polymethoxylated flavones, derived from flavanone in a reaction catalyzed in turn by flavone synthase and flavone A-ring methyl transferases were completely absent in lemon and grapefruit cultivars, suggesting a lower enzyme activity or expression.

### Miscellaneous compounds

**Figure 6:**
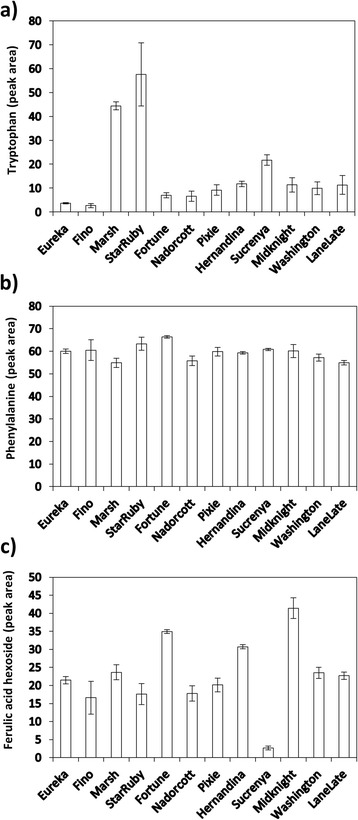
**Relative levels of miscellaneous metabolites identified in citrus juices: tryptophan (a), phenylalanine (b) and a ferulic acid hexoside (c).** Sample ID is given in x-axis.

Precursor compounds such as phenylalanine, ferulic acid hexoside and tryptophan were grouped under this epigraph (Figure 6). Phenylalanine, along with Tyr, is the precursor of all aromatic compounds (among which flavonoids and phenolic acids are found) through reaction catalyzed by PAL and CHS. Results indicated that this precursor compounds are not limiting for all derived compounds and therefore differences in flavonoid composition are due to variations in the expression of genes encoding for metabolic enzymes acting downstream CHS. To this respect, ferulic acid hexoside was scarce in juices of ‘Sucrenya’ but, conversely, this variety did not show any limitation in flavonoid biosynthesis (Figure 5), suggesting that biosynthetic restrains did not affect steps upstream ferulic acid. In the rest of genotypes, this metabolite was moderately abundant with the exception of ‘Fortune’ and ‘Midknight’ which juices had significantly higher levels of this compound. Content of tryptophan, an aminoacid precursor of indolic compounds and the auxin indole-3-acetic acid, was found to be extremely scarce in the vast majority of citrus varieties assayed with the exception of the two grapefruit cultivars studied, with values four-fold higher than the average levels.

## Conclusions

Organoleptic quality is associated not only to primary attributes such as soluble solids (sugars) and acids (mainly citric acid) but other minor compounds such as triterpenoids, flavonoids, coumarins and anthocyanidins. Recently, these compounds have gained scientific and commercial attention due to their beneficial effects on human health and also as important phylogenetic markers. It is well known that metabolites are the downstream products of gene expression and, as such, subjected to a thorough selection process. Therefore, secondary metabolites can be used either as quality traits or as markers for the selection and/or certification of different fruit sources [12] or for the physiological evaluation of plant genotypes [7]. To this regard, in this work we have focused only in commercial subspecies arising from reciprocal crosses between different citrus ancestor lines: *Citrus maxima* (pummelo), *Citrus reticulata* (mandarin), *Citrus medica* (citron) and *Citrus aurantifolia* (mexican lime). To further investigate the inheritance of specific metabolite traits, an exhaustive analysis including these ancestor species should be performed. Nevertheless, the results presented in this manuscript indicate that LC/ESI-QTOF-MS non-targeted metabolite profiling is an efficient technique to profile secondary metabolites in citrus juices with little sample processing (squeezing, centrifuging and filtering). In addition, this technique could be coupled to multivariate analysis as data mining technique to allow separation of different fruit sources: lemons, grapefruits, mandarins, navel and blonde oranges and, more importantly, the differentiation of varieties within a particular group.

## Additional files

Additional file 1: Figure S1. Phylogenetic tree depicting relationships between all known parental ancestor lines and commercial genotypes. Additional file 2: Figure S2. 2D scores (left) and loadings (right) plots depicting different projections: a) component 1 vs. component 2, b) component 2 vs. component 3, c) component 5 vs. component 6, d) component 7 vs. component 8, e) component 9 vs. component 10 and f) component 1 vs. component 11. In scores plots, clearly demarcated variety sample groups are indicated; in loadings plots variables potentially contributing to variety demarcation are indicated in red. Additional file 3: Table S1. Confirmation of Metabolite Candidates for Classification of Citrus Varieties by ANOVA followed by Fisher Least Significant Difference test.

## Abbreviations

LC/MS: Liquid chromatography coupled to mass spectrometry
PTFE: Polytetrafluoroethylene, teflon
QTOF-MS: Quadrupole time-of-flight mass spectrometry
HPLC: High performance liquid chromatography
ESI: Electrospray ionization
HCA: Hierarchical cluster analysis
PLS-DA: Partial least squares discriminant analysis
VIP: Variable importance for the projection (related to PLS-DA)
ABA: Abscisic acid
NCED: Nine cis-epoxycarotenoid dioxygenase
ANOVA: Analysis of variance
PA: Phaseic acid
DPA: Dehydrophaseic acid
ABAGE: Abscisic acid glycosyl ester
PAL: Phenylalanine ammonia lyase
CHS: Chalcone synthase
