# Supplementary material for: Non-targeted metabolite profiling of citrus juices as a tool for variety discrimination and metabolite flow analysis
Source: BMC Plant Biol. 2015 Feb 5;15:38. doi: 10.1186/s12870-015-0430-8 (PMC4329192; doi:10.1186/s12870-015-0430-8)
Supplement: Additional file 1: Figure S1. — Phylogenetic tree depicting relationships between all known parental ancestor lines and commercial genotypes. [file 12870_2015_430_MOESM1_ESM.pdf]

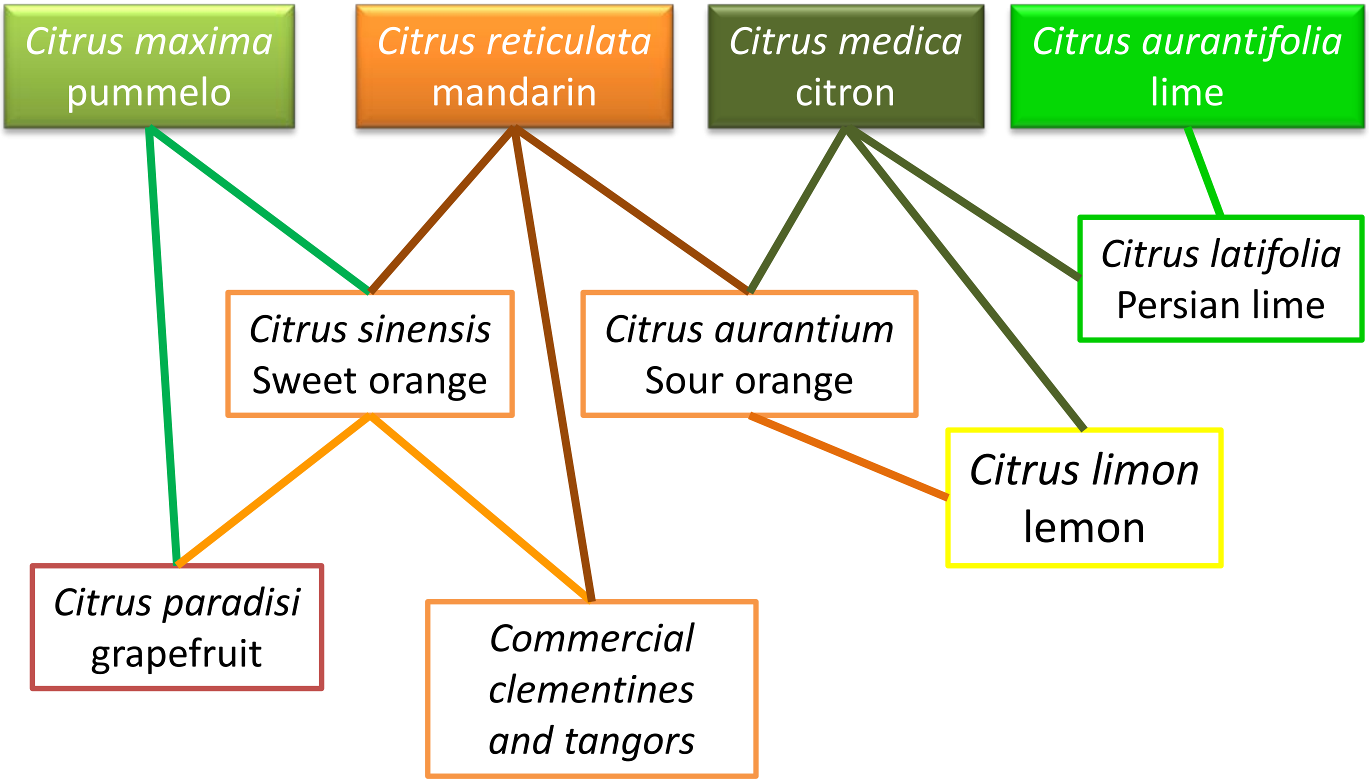

Supplementary Figure 1. Phylogenetic tree depicting relationships between all known parental ancestor lines and commercial genotypes.
