# Supplementary figures and images for: Non-targeted metabolite profiling of citrus juices as a tool for variety discrimination and metabolite flow analysis
Source: BMC Plant Biol. 2015 Feb 5;15:38. doi: 10.1186/s12870-015-0430-8 (PMC4329192; doi:10.1186/s12870-015-0430-8)

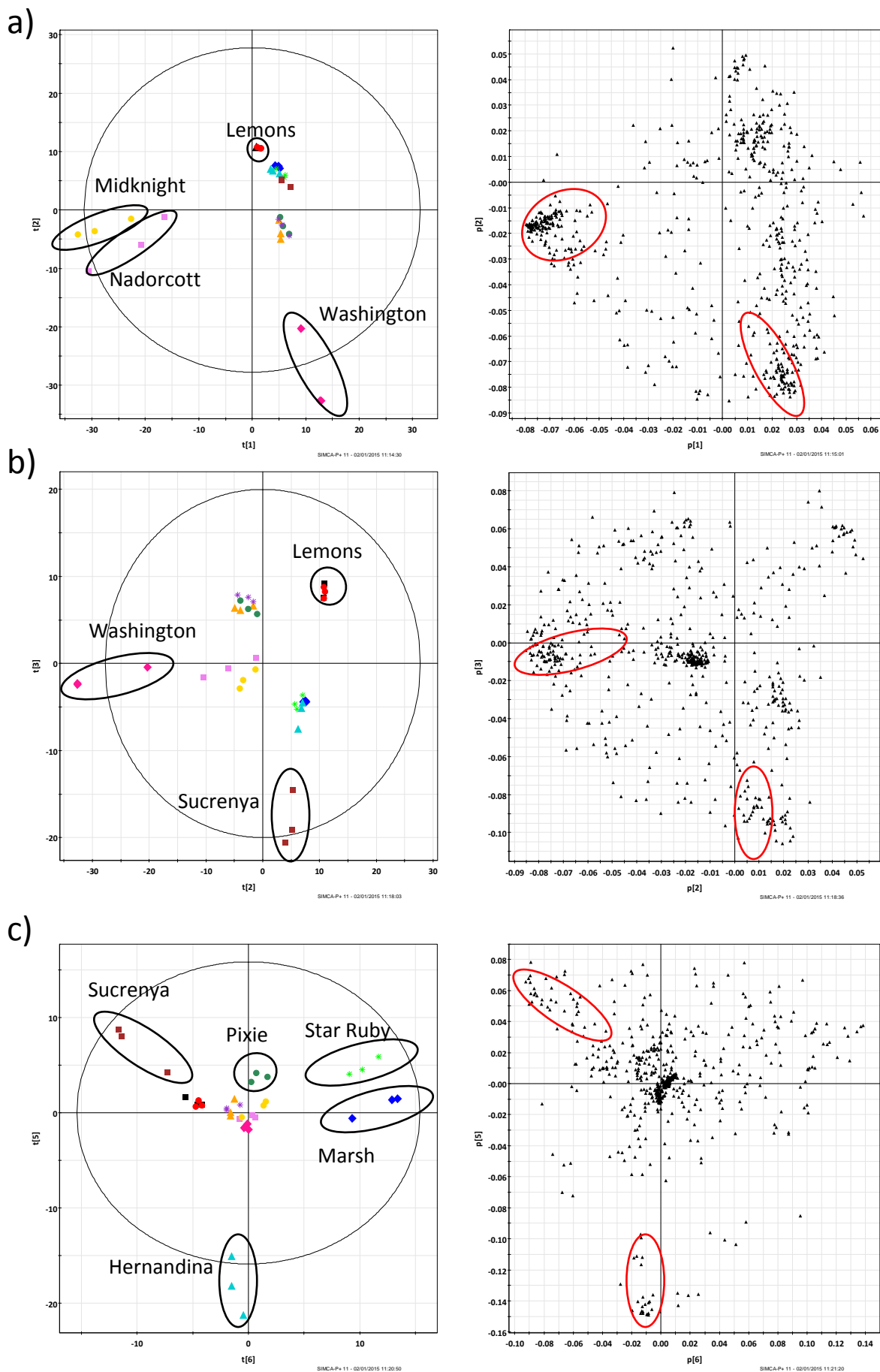

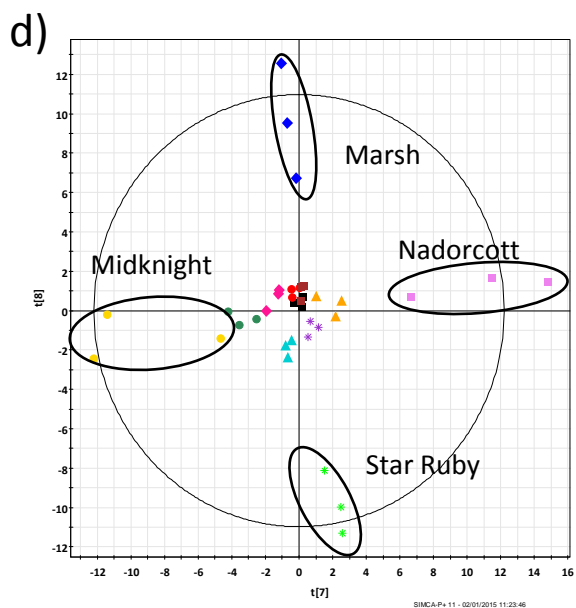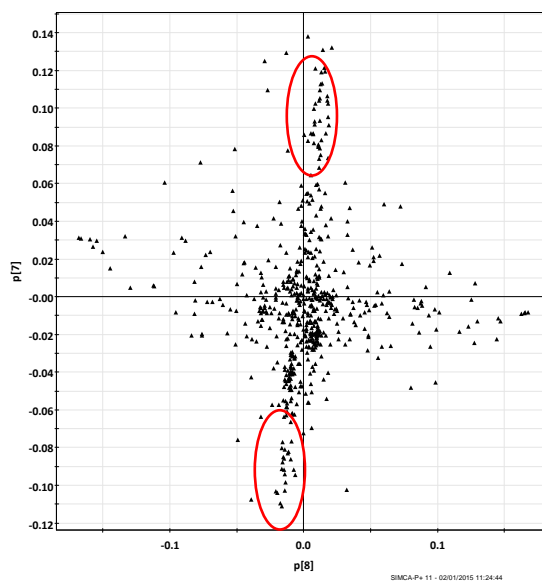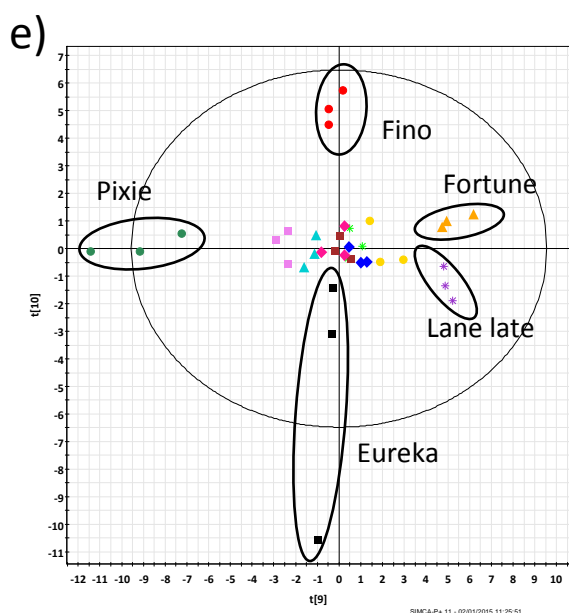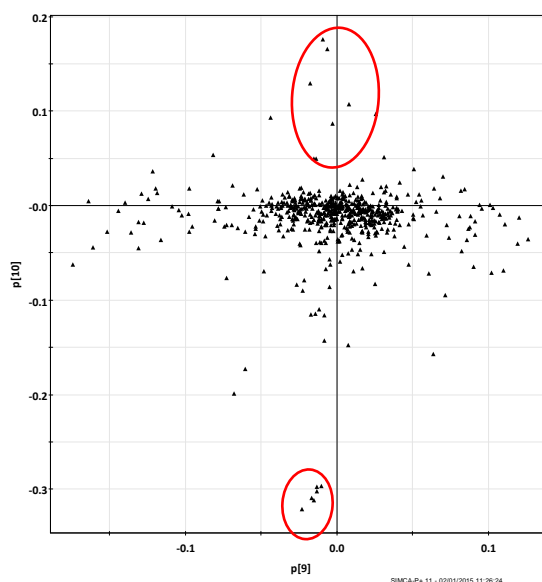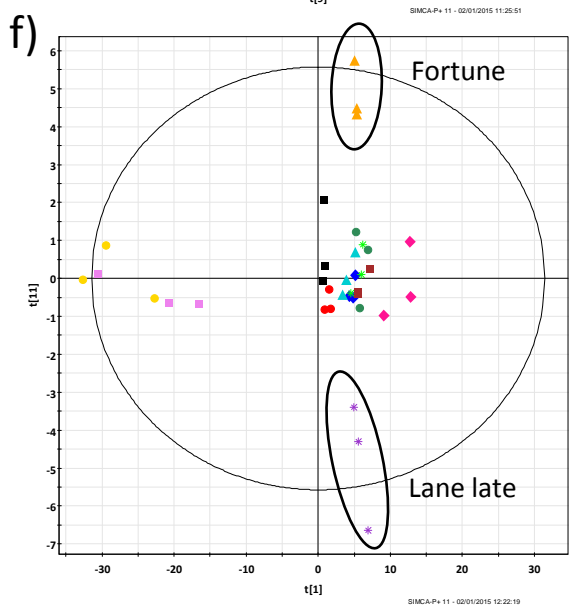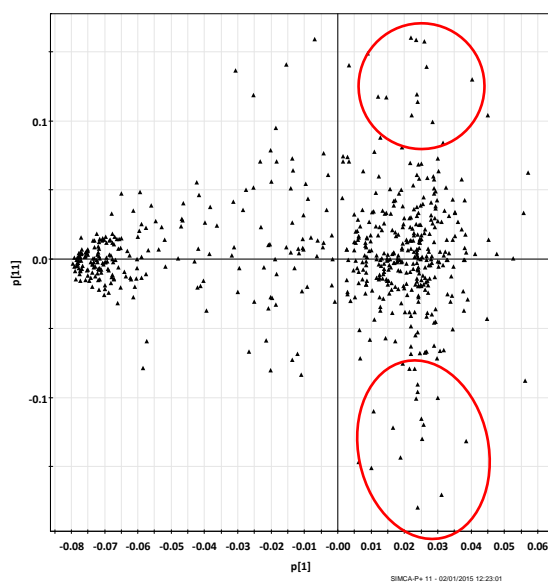

Supplementary Figure 2. continued.

Supplement: Additional file 2: Figure S2. — 2D scores (left) and loadings (right) plots depicting different projections: a) component 1 vs. component 2, b) component 2 vs. component 3, c) component 5 vs. component 6, d) component 7 vs. component 8, e) component 9 vs. component 10 and f) component 1 vs. component 11. In scores plots, clearly demarcated variety sample groups are indicated; in loadings plots variables potentially contributing to variety demarcation are indicated in red. [file 12870_2015_430_MOESM2_ESM.pdf]
